# Protocol for culturing olfactory epithelium organoids supporting neuronal differentiation

**DOI:** 10.1016/j.xpro.2025.104211

**Published:** 2025-11-14

**Authors:** Juliana Gutschow Gameiro, Jussi Virtanen, Liam Lee, James E. Schwob, Marco Aurélio Fornazieri, Brian Lin

**Affiliations:** 1 Department of Clinical Surgery, State University of Londrina, Paraná, Londrina, Brazil; 2 Department of Developmental, Molecular and Chemical Biology, Tufts University School of Medicine, and Graduate School of Biomedical Sciences, Boston, MA 02111, USA; 3 Department of Otorhinolaryngology, Head and Neck Surgery, Tampere University Hospital, Tampere, Finland; 4 Faculty of Medicine and Health Technology, Tampere University, Tampere, Finland; 5 Department of Medicine, Pontifical Catholic University of Paraná, Londrina, Brazil; 6 Health Sciences Graduate Program, State University of Londrina, Paraná, Londrina, Brazil

**Keywords:** Cell culture, Cell isolation, Developmental biology, Stem Cells, Organoids

## Abstract

The olfactory epithelium (OE) contains stem cells capable of generating olfactory sensory neurons throughout life, making it a valuable model for studying epithelial neurogenesis. We present a protocol to develop a highly robust 3D mouse organoid model from OE cells. We describe a workflow for dissecting murine OE and subsequent organoid culturing. We also provide guidance on how to perform immunostaining of the organoids. This protocol has been validated for mice and does not require fluorescence-activated cell purification.

For complete details on the use and execution of this protocol, please refer to Gameiro et al.^1^

## Highlights

• Instructions for mouse olfactory epithelium harvesting
• Steps for developing a 3D but adherent mouse olfactory epithelium organoids
• Guidance for immunostaining of olfactory epithelium organoids

## Abstract (editor highlights)

Publisher’s note: Undertaking any experimental protocol requires adherence to local institutional guidelines for laboratory safety and ethics.

## Graphical abstract

**Figure:**
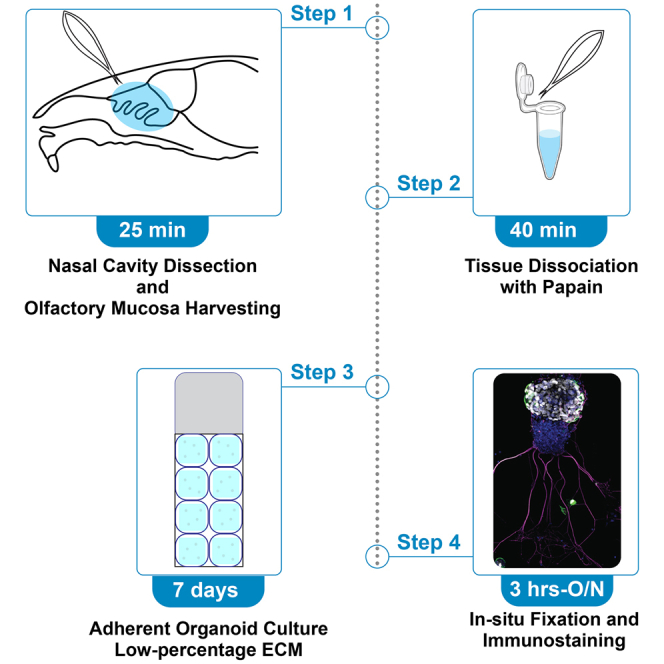

## Before you begin

1. See the full list of reagents in the key resources table.
2. Prepare equipment for dissection (Figure 1).

**Figure 1:**
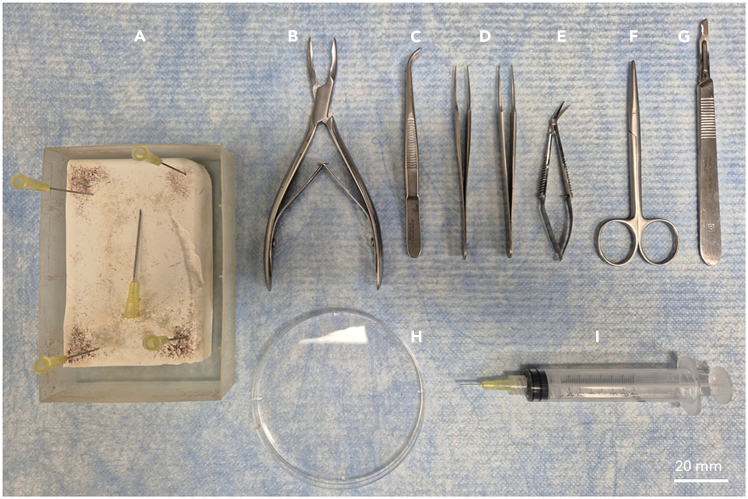
Equipment for the olfactory epithelium dissection (A) A home-made dissection board and five 20G needles (0.9 mm x 40 mm) (BD PrecisionGlide) to fix the mouse for dissection. (B) Pearson Rongeurs (Fine Science Tools). (C) Narrow pattern forceps (Fine Science Tools). (D) Two Dumont #5 Fine Forceps (Jeweller’s forceps) (Fine Science Tools). (E) Spring scissors (Fine Science Tools). (F) (Strabismus) Scissors (Fine Science Tools). (G) Scalpel #15 blade (Fine Science Tools). (H) 10 cm Petri dish. (I) 10 ml syringe (BD Luer-Lok) and 20G needle (0.9 mm x 40 mm) cut and blunted manually (BD PrecisionGlide).
3. Ensure all enzymatic reagents and growth media are thawed on ice or prepared fresh on the day of the experiment and kept on ice.
  a. Papain dissociation mix.
  b. Organoid growth media.
4. Cultrex-containing media must be kept on ice at all times to prevent premature solidification.

### Innovation

This method describes a facile method for growing mouse olfactory organoids in vitro, eliminating the need for transgenic animals or purification steps beyond anatomical dissection. Furthermore, we describe a cost-effective method for growing olfactory organoids that are attached to a flat plastic or glass substrate, facilitating handling and imaging.

### Institutional permissions

The protocol requires the use of animals. Governmental and institutional guidelines should be followed during all animal experiments. In this protocol, all mice were maintained following Institutional Animal Care and Use Committee (IACUC) guidelines, with ad libitum rodent chow and water. The mice were housed in rooms with controlled heat (20°C–24.4°C) and humidity (30°C–70% relative humidity), and a 12:12 h light-dark cycle. The committee approved all procedures using vertebrate animals for the Humane Use of Animals at Tufts University School of Medicine under protocol B2023-98.

## Key resources table

**Table:** 

REAGENT or RESOURCE	SOURCE	IDENTIFIER
**Antibodies**		

Rat anti-Sox2, 1:500	eBioscience, Invitrogen	RRID: AB_2865466; Cat# 501129095
Chicken anti-Krt14, 1:500	BioLegend	RRID: AB_2616962; Cat# 906004
Mouse anti-TUBB3 (Tuj1), 1:1,000	BioLegend	RRID: AB_2313773; Cat# 801202

**Chemicals, peptides, and recombinant proteins**		

DAPI	Thermo Fisher Scientific	Cat# D3571
HBSS 1X	Thermo Fisher Scientific	Cat# 14175095
Papain	Worthington Biochemical	Cat# LS003119
DNase I	Worthington Biochemical	Cat# LS002139
DMEM/F12	Thermo Fisher Scientific	Cat# 1132033
Primocin 1x	InvivoGen	Cat# ant-pm
Recombinant murine R-spondin 1	Thermo Fisher Scientific	Cat# 315-32
Recombinant murine Noggin	Thermo Fisher Scientific	Cat# 250-38
Recombinant murine Wnt-3a	Thermo Fisher Scientific	Cat# 315-32
Y27632 (10 μM)	Tocris Bioscience	Cat# 1254
N2 100x	Thermo Fisher Scientific	Cat# 17502048
B27 50x	Thermo Fisher Scientific	Cat# 17504044
GlutaMAX 100x	Thermo Fisher Scientific	Cat# 35050061
HEPES 1M	Thermo Fisher Scientific	Cat# 15630080
Cultrex Stem Cell Qualified Reduced Growth Factor Basement Membrane Extract	Bio-techne	Cat# 3434-005-02
Trypan blue	Thermo Fisher Scientific	Cat# 15250061
PBS pH 7.4 1x	Thermo Fisher Scientific	Cat# 10010023
Bovine serum albumin (BSA)	Fisher Scientific	Cat# BP1600-100
Tween 20	MilliporeSigma	Cat# P9416
Triton X-100	MilliporeSigma	Cat# T8787

**Other**		

A dissection board	Homemade	Poured using Sylgard 184
20G needle (0.9 mm × 40 mm)	BD PrecisionGlide	Cat# 305176
20G needle (0.9 mm × 40 mm) cut and blunted manually	BD PrecisionGlide	Cat# 305176
10 mL syringe	BD Luer-Lok	Cat# 302995
Pearson Rongeurs	Fine Science Tools (FST)	Cat# 16015-17
Scalpel handle	FST	Cat# 10003-12
Scalpel #15 blade	FST	Cat# 10015-00
Two Dumont #5 Fine forceps (Jeweller’s forceps)	FST	Cat# 11254-20
Narrow pattern forceps	FST	Cat# 11003-12
(Strabismus) Scissors	FST	Cat# 14074-11
Spring scissors	FST	Cat# 15006-09
Safe-Lock tubes 1.5 mL - microtube	Eppendorf	Cat# 05-402-25
15 ml conical tubes	Fisher Scientific	Cat# 22-171-716
8-well chamber well	NEST	Cat# NST230108
Replaceable Polystyrene microscope slides, TC-treated	Evergreensci Caplugs	Cat# 333-5689-01T

## Materials and equipment

**Table:** Papain dissociation mix

Reagent	Final concentration	Amount
HBSS	N/A	750 uL
Papain (dissolve in HBSS to a stock 40 U/ml)	10 U/mL	250 uL
DNaseI (dissolve in HBSS to a stock 75 kU/ml)	15 U/mL	2 uL
**Total**	**N/A**	**∼1 ml**

***Note:*** Lyophilized papain from Worthington Biochemical includes instructions for activation with β-mercaptoethanol and cysteine-HCl. In our hands, these additives did not affect dissociation efficiency, and we therefore omit them.

***Note:*** Papain and DNase may be frozen in aliquots at −80°C for up to 6 months. Prepare the required amount of dissociation mix fresh for each dissection; do not store the mix.

**Table:** Organoid growth media with Cultrex

Reagent	Final concentration	Amount
DMEM/F12 with Primocin 1x	N/A	4.5 mL
Recombinant murine R-spondin 1 (dissolve in sterile ddH2O to a stock of 0.1 mg/ml)	200 ng/mL	10 uL
Recombinant murine Noggin (dissolve in sterile ddH2O to a stock of 0.1 mg/ml)	100 ng/mL	5 uL
Recombinant murine Wnt-3a (dissolve in sterile ddH2O to a stock of 0.1 mg/ml)	50 ng/mL	2.5 uL
Y27632 (dissolve in ddH2O to a stock of 5 mM)	10 μM	10 uL
N2	1%	50 uL
B27	2%	100 uL
GlutaMAX 100x	1%	50 uL
HEPES 1M	10 mM	50 uL
Cultrex Stem Cell Qualified Reduced Growth Factor Basement Membrane Extract	4% vol/vol	200 uL
**Total**	**N/A**	**5 mL**

***Note:*** Reconstitute R-spondin, Noggin, Wnt-3a, and Y27632 in ddH2O and aliquot into individual single-use tubes. We recommend storing in −80°C out of an abundance of caution, but −20°C should be sufficient for at least 1 year.

**CRITICAL:** Nutrient-rich media is susceptible to fungal contamination, which can impair organoid growth. Use Primocin or another antibiotic with anti-mycotic activity; do not use Pen/Strep or antibacterials lacking anti-mycotic coverage. Keep media cold to prevent premature gelling; media is stable up to 7 days.

**Table:** Organoid growth media without Cultrex

Reagent	Final concentration	Amount
DMEM/F12 with Primocin 1x	N/A	4.7 mL
Recombinant murine R-spondin 1 (dissolve in sterile ddH2O to a stock of 0.1 mg/ml)	200 ng/mL	10 uL
Recombinant murine Noggin (dissolve in sterile ddH2O to a stock of 0.1 mg/ml)	100 ng/mL	5 uL
Recombinant murine Wnt-3a (dissolve in sterile ddH2O to a stock of 0.1 mg/ml)	50 ng/mL	2.5 uL
Y27632 (dissolve in ddH2O to a stock of 5 mM)	10 μM	10 uL
N2	1%	50 uL
B27	2%	100 uL
GlutaMAX 100x	1%	50 uL
HEPES 1M	10 mM	50 uL
**Total**	**N/A**	**5 mL**

**CRITICAL:** Nutrient-rich media is susceptible to fungal and mycoplasma contamination, which can impair organoid growth. Use Primocin or another antibiotic with anti-mycotic activity; do not use Pen/Strep or antibacterials lacking anti-mycotic coverage. Keep media cold to prevent premature gelling; media is stable up to 7 days.

**Table:** Wash buffer (PBST) for immunostaining

Reagent	Final concentration	Amount
PBS 1x	N/A	100 mL
Tween 20	0.05%	5 uL
**Total**	**N/A**	100 mL

***Note:*** PBST has a tendency to become contaminated with microbial growth when stored at room temperature. We recommend storing this at 4°C, making it stable for at least 3 months.

**Table:** Antibody dilution buffer for immunostaining

Reagent	Final concentration	Amount
PBS 1x	N/A	50 mL
BSA	1%	0.5 g
Triton X-100	0.3%	150 uL
**Total**	**N/A**	**50 mL**

***Note:*** Antibody dilution buffer contains BSA, rendering it very susceptible to microbial growth. Store at 4°C and, preferably, on ice when handling to increase shelf-life. Kept this way, it is stable for at least 1 month.

## Step-by-step method details

### Olfactory mucosa harvesting

**Timing: 25 min**

This section describes how to harvest the olfactory mucosa of the 5–8 week old male or female mouse. The technique maximizes intact tissue and avoids inclusion of respiratory epithelium.

***Note:*** The above timing equals the time required to do the olfactory mucosa harvesting of one mouse.

**Figure 2:**
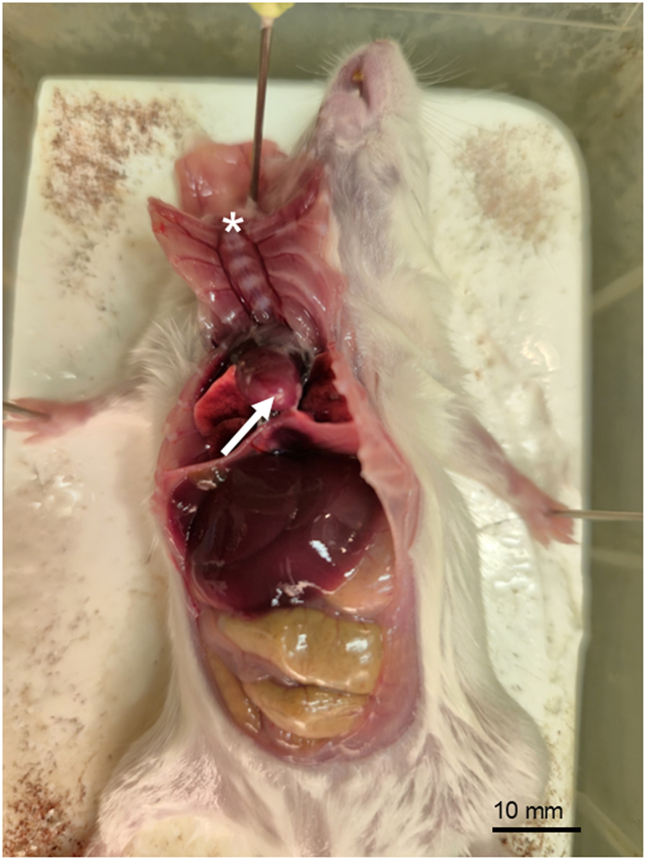
Preparing for transcardiac flushing The thorax was opened, and the heart exposed. The thorax and skin are attached to the dissection board with a needle (asterisk). A small incision should be made to the right atrium (arrowheads). Another small incision should be made to the apex of the heart (arrow) for the insertion of a perfusion needle.
  c. Grab the heart with narrow pattern forceps and make small cuts with spring scissors to the right atrium and left ventricle of the heart (Figure 2).
  d. Insert a blunt needle into the left ventricle and flush the circulation.

***Note:*** You should not feel resistance during flushing.

1. Use a carbon dioxide (CO_2_) inhalation chamber to sacrifice 5-8 week old male or female mice until no movement is noticed.
2. Perform transcardiac flush with 10 ml of PBS (pH 7.4, 1x) to remove blood.
  a. Affix the mouse on the dissection board from the limbs using 20G needles. (Styrofoam block can also be used)
  b. Use scissors to cut through the skin and peritoneum.
    i. Move the liver out of the way and cut through the diaphragm.
    ii. Cut along the mid-thoracic plane on both sides to expose the heart.
    iii. Use one needle to attach the skin and the detached part of the thoracic wall to the foam bed next to the mouse head (Figure 2).

**CRITICAL:** The liver turns pale during flushing, proving that the needle is placed correctly. If fluid comes out of the mouth or the nostrils, adjust the needle position. If PBS leaks out of the incision, next time make a smaller nick. Clamping with forceps can help reduce leakage.

3. Decapitate the mouse.

***Note:*** At this point, you can put the decapitated head on ice and do the steps above on another mouse. We process a maximum of three mice at one time. A decapitated head can stay on ice for approximately 30 min. If a longer interval is expected, the recommendation is to proceed to fine dissection before processing the next animal.

4. Peel the skin over the head.
  a. Use a 15-blade to make a midline incision from the back of the skull all the way to the tip of the nose (Figure 3A).

**Figure 3:**
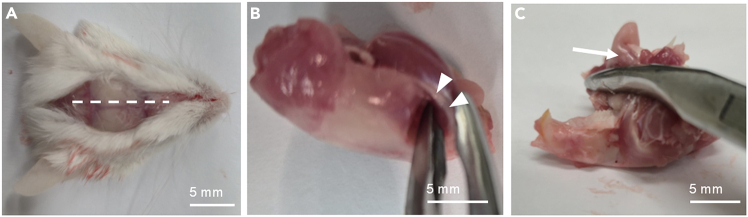
Removal of the soft tissue of the skull After decapitating the mouse, a midline longitudinal incision (dotted line) is made (A). The skull is turned sideways, and the zygomatic bones (arrowheads) are crushed on both sides with rongeurs (B). The skull is turned upside down, and the tongue and the jaw (arrow) are removed with rongeurs by pulling them posteriorly (C).
  b. Remove the skin by pulling it by hand, and once reaching the tip of the nose, pull the skin away.
5. Use rongeurs to remove the eyeballs and to break the zygomatic bones covering the orbits (Figure 3B).
6. Break the incisors with rongeurs.
7. Turn the head upside down and grab the tongue and the lower jaw with rongeurs and pull hard posteriorly to remove them (Figure 3C).
8. Remove soft tissue (muscle, tendons) with rongeurs to expose the bony skull completely (Figure 4A).

**Figure 4:**
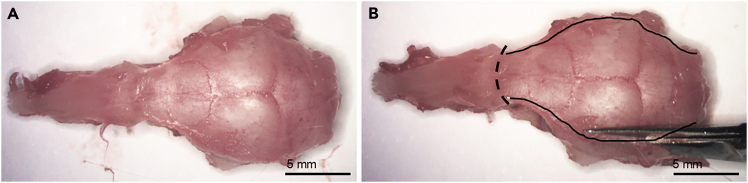
Exposure of the brain and the olfactory bulbs Remaining soft tissue is removed to expose the bony skull (A). The bones of the skull are removed all the way up to the frontonasal suture (curved line) to expose the brain and the olfactory bulbs. Drawn lines show where to make the cuts (B).
9. Use small spring scissors to remove the bones covering the skull.
  a. Insert one blade of the scissors via the foramen magnum under the occipital bone posteriorly and the other blade on top of the bone.
  b. Cut the bone on both sides, posteriorly to anteriorly all the way to the frontonasal suture (Figure 4B).
  c. The bones can now be lifted and removed in one or two pieces using rongeurs or scissors to expose the brain and olfactory bulbs.

***Note:*** Sometimes a small piece of frontal bone remains posterior to the frontonasal suture (and nasal bone). This can be removed with forceps.

***Note:*** From now on, the dissection can be performed under the microscope or with the naked eye, depending on personal preference.

10. Remove the posterior part of the maxillary bones.
  a. Using spring scissors, cut the maxillary bone on both sides, beginning dorsally and extending to the molar teeth. The cut is positioned between the olfactory bulb and the brain (Figure 5A).

**Figure 5:**
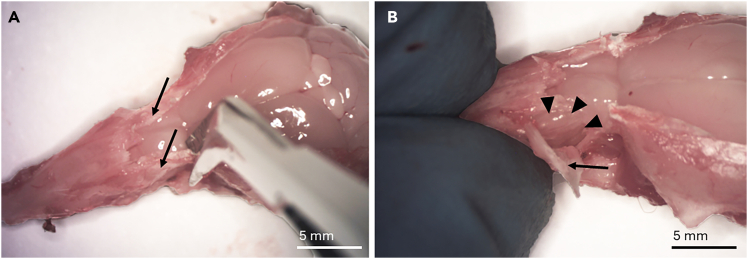
Removal of the maxillary bone The maxillary bones on both sides (arrows) are removed by making a vertical incision with spring scissors through the maxillary bone to separate it from the skull. The place for the incision (dotted line) is between the olfactory bulb (asterisk) and the brain (square) (A). Then, the maxillary bone (arrow) is lifted carefully to keep the olfactory turbinate block (arrowheads) intact (B).
  b. Use forceps to pry off (or pull away) maxillary bones.
    **CRITICAL:** Remove the bone carefully so that you do not remove or damage the turbinates. Use of fine forceps (jeweler's forceps) is best here, though ones with duller/blunt ends are preferred. If forceps are too new and sharp, they will cut through the bone instead of gripping it properly (Figure 5B). If this becomes problematic, a dedicated pair of forceps can be dulled purposefully on a rough edge and then aligned using a whetstone.
  c. Now you should be able to visualize the cribriform plate and lateral aspect of the olfactory turbinate block.
11. Remove the nasal bones.
  a. Grab the tip of the nose with fingers or forceps and use other forceps to lift the nasal bones in a posterior-to-anterior direction (Figure 6A).

**Figure 6:**
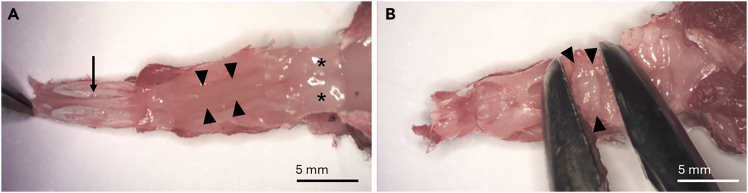
Removal of the nasal bone and the mucosa covering the hard palate The nasal bone (arrow) is lifted with forceps. The mucosa of the nasal septum (arrowheads) and the olfactory bulbs (asterisk) are also visible (A). The hard palate (arrowheads) is removed by pulling it posteriorly using rongeurs (B).
  b. Cut the nasal bones from the tip of the nose.
12. Remove the brain and the olfactory bulbs by grabbing the olfactory bulbs with forceps and pulling them posteriorly together with the brain.
13. Flip the head upside down and strip the mucosa covering the hard palate (behind incisors) with rongeurs and pull it off (Figure 6B).
14. Crunch molars with rongeurs or cut the jaw above them with spring scissors from the posterior to anterior direction.
15. Remove the maxillary and premaxillary bones.
  a. The remaining incisor teeth are attached to the premaxillary bones on both sides.
  b. Head still upside down, insert one blade of the spring scissors behind the incisors between the premaxillary bone and septum, and the other blade anteriorly to the incisors and cut (Figure 7A).

**Figure 7:**
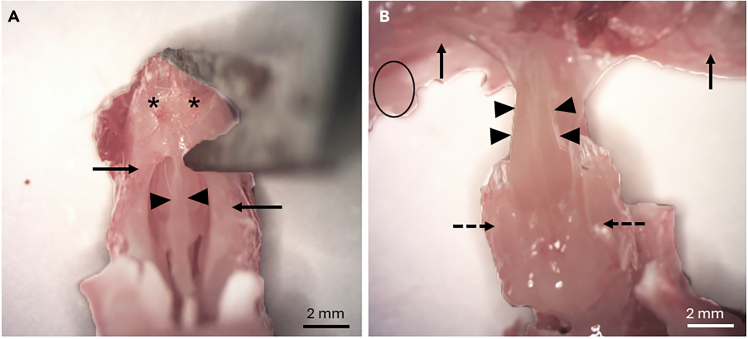
Removal of the premaxillary and maxillary bones The premaxillary bones (arrows) attached to the incisors are separated from the septum (arrowheads) using spring scissors. The other blade of the scissors is placed between the septum and the premaxillary bone, and the other one anteriorly to the remnants of the incisors (asterisk) (A). Finally, the premaxillary (arrows) and maxillary bones (not visible) are removed completely by grasping them posteriorly and pulling in the anterior direction, which removes the parotid glands (circle) and spares the turbinates (dotted arrows) and the mucosa of the septum (arrowheads) (B).
  c. Repeat on the other side.
    ***Note:*** The entire structure becomes loose, so hold it gently with two fingers while cutting premaxillary bones off.
  d. Flip the head back on upright position and use two forceps to remove the rest of the maxillary and premaxillary bones.
  e. Hold the piece with the other forceps and carefully grab the posterior part of the maxillary bone and pull anteriorly with the other forceps. This will remove the bones in one piece and also the parotid gland (Figure 7B).
  f. Repeat on the other side (Figure 7B).
16. Now the septum and the turbinates are exposed (Figure 8A). Remove these in one piece from the skull by cutting with spring scissors.

**Figure 8:**
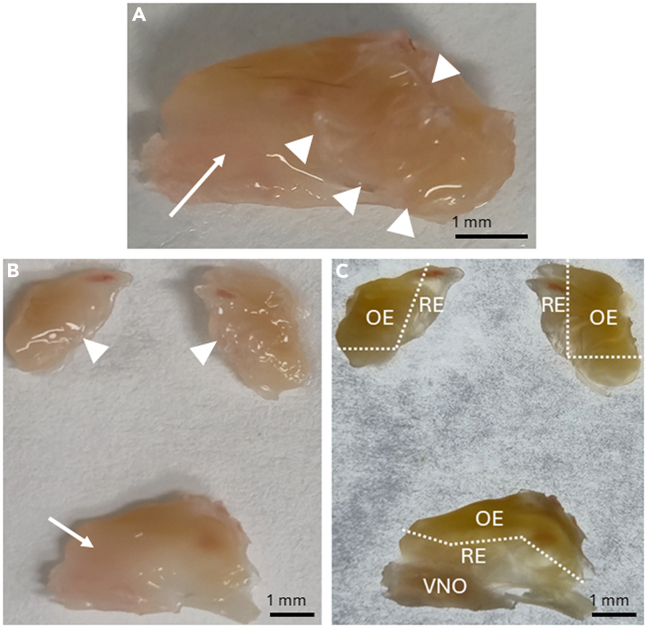
Separating the septum and the turbinates The septum (arrow) and the turbinates (arrowheads) are cut from the skull (A). The spring scissors are inserted between one turbinate and the septum, and the turbinate is released with one or two cuts. The septum (arrow) and medial sides of the turbinates (arrowheads) are visualized (B). The olfactory epithelium (OE) is distinct in color (yellow) compared to the respiratory epithelium (RE), which is clear in color. The RE is removed to keep only the OE. The vomeronasal organ (VNO) is also visible (C).
17. Put the sample containing septum and turbinates on a 10 cm plate with 18°C–22°C 1X PBS covering the plate (not used in the attached figures to improve picture quality).

***Note:*** Limit the amount of PBS used to prevent samples from floating while trying to dissect.

18. Use fine forceps and spring scissors to separate the turbinates from the septum.
  a. Insert one blade of the scissors between the septum and turbinate anteriorly and cut posteriorly to detach the turbinate block (Figure 8B).
    ***Note:*** There should be no resistance if the blade is positioned within the dorsal recess of the nasal cavity. If resistance is felt, back up and adjust the angle slightly.
  b. Repeat on the other side (Figure 8B).
  c. Remove the respiratory epithelium from the turbinates and septum using spring scissors and forceps (Figure 8C).

### Tissue dissociation

**Timing: 40 min**

This section describes how to obtain single cells from the OE tissue harvested from the previous step. By the end, the cells can be used directly in culture after cell counting.

***Note 1:*** After incubation, all steps are done on ice and performed as quickly as possible to minimize cell death. Pipette gently.

***Note 2:*** The quantities below are equivalent to 1 mouse OE. If more animals are needed, increase the volumes proportionally and use bigger tubes or process each sample individually (remember to maintain adequate air space to facilitate proper mixing).

19. Place the dissected OE tissue to the cap of a 1.5 mL microtube and mince into fine pieces using spring scissors until the bone is fully fragmented and no more crunching of bones is felt.

***Note:*** The tissue is minced in the cap for maximal preservation of cell yield as it allows all tissue to be immediately immersed in the papain mix.

20. Add the papain dissociation mix to the tube and close the top.
21. Invert until thoroughly mixed.
22. Incubate for 15 min with gentle agitation (tilt-rocker) at 37°C.
23. After 15 min, pause the incubation.
24. Gently pipette up and down (with a large-bore pipette tip or cut-edge tip) the liquid to dissolve the entanglement formed with the bones and tissues.

**CRITICAL:** Gently pipetting the sample after the first 15 min of incubation will improve the overall cell yield.

25. Resume incubation for another 15 min with gentle agitation (tilt-rocker) at 37°C.
26. Allow the tissue to settle naturally at the bottom of the tube for 3–5 min, then carefully aspirate the supernatant and transfer it to a 15 mL conical tube.
27. Add 5 mL of HBSS 1x to make up a total of about 6 mL.
28. Centrifuge at 500g for 5 min in a swinging bucket rotor at 22°C.
29. Remove the supernatant, and be careful not to aspirate or disintegrate the pellet.

***Note:*** If pellet accidentally becomes disintegrated, do the centrifugation again at the same settings.

30. Resuspend the pellet in 1 mL of DMEM/F12 with 1x primocin.

### Organoid culture

**Timing: 1 week**

This section describes how to culture the OE cells into organoids using chamber slides. By the end of the 7th day, you should see organoids, which appear as balls of cells adhered to the bottom of the well, forming multilayered structures with projections extending from them.

**CRITICAL:** Media containing Cultrex must be kept on ice at all times, as it will prematurely solidify and cannot be used again. Do not pre-warm the media or the chamber-wells before plating to avoid the premature solidification mentioned before.

***Note:*** Matrigel is an alternative for Cultrex; however, the percentage used needs to be recalibrated based on the concentration of the Matrigel batch.

***Note:*** B27 can be obtained with or without retinoic acid. There was no observable difference in capability for neuronal differentiation.

***Note:*** Incubate cells at 37°C in a humidified incubator with 5% CO2.

31. Take 5μL of the resuspended sample and mix it with 5μL of trypan blue.
  a. Apply 10 μL of the cell/trypan blue mixture to the hemocytometer. Count the number of viable (non-blue) cells within the central 5 × 5 grid.
  b. Multiply that number by 20 to get the number of cells/μL.

***Note:*** In a hemocytometer, the central 5 × 5 grid has a defined volume of 0.1 μL. Thus, the number of viable cells counted in this grid corresponds to cells/0.1 μL. Multiplying by 10 gives cells/μL. Because the cell suspension was mixed 1:1 with trypan blue, an additional factor of 2 is applied, resulting in a total conversion factor of ×20.

**Figure 9:**
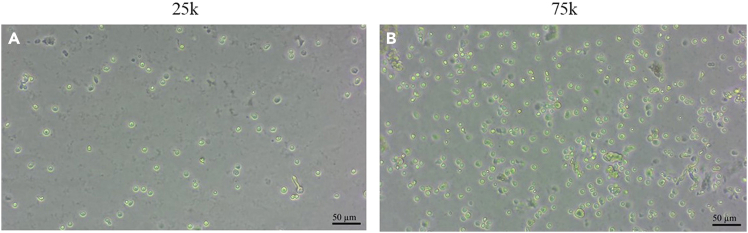
Seeding density Comparison of (A) 25k vs (B) 75k cells seeded in an 8-well chamber slide.
34. Incubate in standard mammalian culture conditions, at 37°C with 5% CO2 in saturated humidity.
35. The next day, gently add 200 μL of media (without Cultrex) down the side of each well to avoid disturbing the existing layer. By this point, the Cultrex has already solidified, as it gels within ∼30 min at 37°C.

***Note:*** Growth media containing Cultrex is only required during initial plating for organoid formation; omitting Cultrex from subsequent media changes is cost-effective and does not affect culture quality.

**Figure 10:**
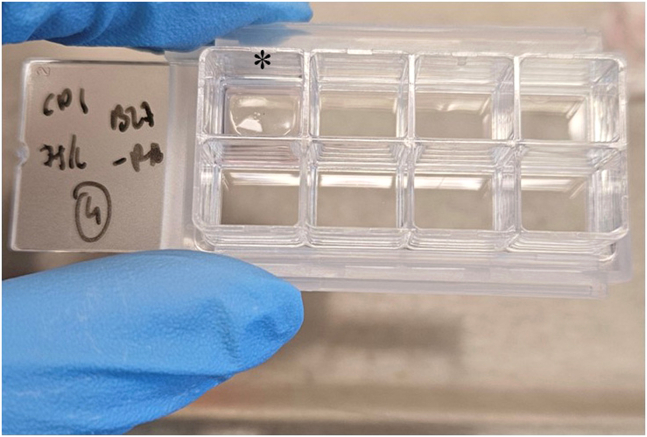
Example chamber well with Cultrex coating Asterix marks the well that has been aspirated, revealing a shiny layer of extracellular matrix, with a small meniscus of media left behind to prevent aspiration of the cells.

**Figure 11:**
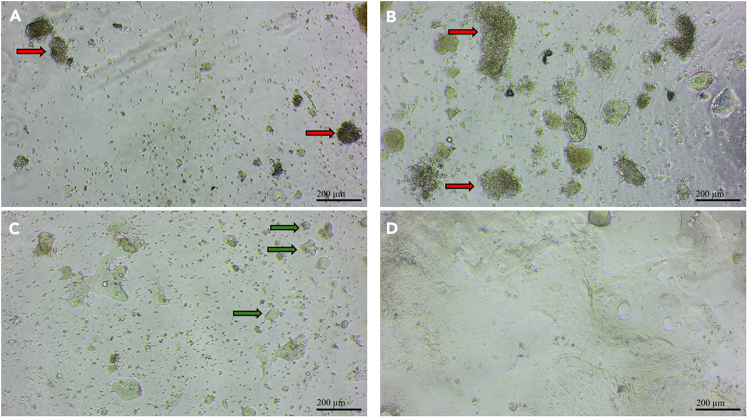
Light microscopy images on different days Red arrow = Debris; Green arrow = Putative organoid. (A) 1 day after seeding. Primarily individual cells and debris from dissection. (B) 2 days after seeding, before aspiration of media so there are more debris (C) 4 days after seeding. Less debris, more hollow-looking spheres. Some formation of a mat of cells. (D) 7 days after seeding. The majority of the area is covered by a mat of cells. which can obscure the organoid structures and make them less distinct on light microscopy.

32. Seed 75k cells per well of an 8-well chamber slide. Add the cell suspension in DMEM/F12 to 200 μL of media already in the well.
  a. Final volume of media (200 μL) with addition of suspended cells in DMEM/F12 should be no more than 220 μL to minimize diluting the nutrients in the media.
  b. If the volume of cells needed to achieve 75k cells/well is above 20 μL, concentrate the cells by centrifugation.
  c. To concentrate, calculate the total number of cells needed, multiply by 1.25 (to compensate for lost cells), spin down the pellet, as before, at 500g for 5 min at 22°C, but resuspend in the exact volume of complete growth media needed. Minimize bubbles when resuspending.
33. Plate cells into room temperature chamber slides at 75k cells/well (Figure 9A–9C, 25k vs 75k density comparison)

36. Aspirate media for the first time after 2 days of plating, and then every other day Figure 10)
  a. Aspirate the media with a 200μL tip very carefully and slowly. You will see a shiny layer of Cultrex at the bottom of the well–do not remove it since your cells are in it.
    ***Note:*** If media is replaced on a Friday, the culture can be left over the weekend, and a media change can be done on Monday.
    ***Note:*** Most chamber slides have a plastic seam along the sidewalls that can be used as a safety landmark. During aspiration or media changes, keep the pipette tip above this seam to avoid accidentally disturbing or aspirating the underlying gel.
  b. Add 200 μL of growth media without Cultrex directly to the well, slowly.
    ***Note:*** It is possible to observe initial projections in some organoids by day 5, but full extension should occur by day 7.
    ***Note:*** True organoids can be distinguished from tissue debris on light microscopy based on morphology. A true organoid will appear more spherical with a hollow-looking center, in contrast to tissue debris, which will have irregular borders and often be floating/mobile. (Figure 11, *light microscopy images*)
    ***Note:*** Culture can be maintained for up to 14 days. Beyond this point, organoid health declines, as cells begin to lose adhesion and detach into the medium. We recommend staining on day 7 for optimal results.

### Fixation and immunostaining

**Timing: 3 h to overnight**

This section describes how to fix the organoids and immunostain for the primary markers expressed by the cells present in the organoids.

***Note:*** Organoids can be grown in normal tissue-culture treated plastic wells, but growing them in chamber slides may help facilitate analysis by immunofluorescence and maintains their morphology. This is because the organoids can be imaged directly in the same well, without the need to detach them and transfer to a separate imaging plate.

***Note:*** The pH of the 4% PFA must be adjusted to pH 7.2–7.4 before use. If pH is not within this range, it will dissolve the matrix-gel structure, and organoids will be lost. You can store pH adjusted aliquots frozen in −20°C, but do not reuse them after thawing.

37. On day 7 of culture, wash and fix the wells.
  a. Aspirate the media completely, leaving only the gel coating behind. Washing with PBS is optional at this point.
  b. Add 4% PFA (prepared in 1x PBS, pH 7.2–7.4) directly to the wells for 15 min at room temperature.
  c. Remove the 4% PFA carefully.
    ***Note:*** The shiny, gelatinous surface should no longer be visible, and it should be possible to see the larger and eye-visible organoids attached to the chamber well bottom.
  d. Incubate with PBST for 15 min.
    **CRITICAL:** Incubation with PBST is required for adequate permeabilization for staining.
38. Prepare primary and secondary antibody solutions. The concentration of antibodies used and the cells that they label are listed under key resources table.
  ***Note:*** Be attentive to the type of detergent used in antibody dilution buffer. In the antibody dilution buffer, use Triton X-100, not Tween 20 as used in PBST.
  ***Note:*** A separate blocking step is not necessary for staining these organoids, as the antibody dilution buffer also acts a blocking buffer.
  a. Incubate samples in primary antibodies for 1 h at RT or overnight at 4°C with gentle agitation.
    ***Note:*** DAPI counterstain can be added at either the primary or secondary antibody incubation step.
  b. Wash 3 times with PBST after each incubation.
  c. Incubate in secondary antibodies for an hour at RT.
  d. Wash 1-3 times with PBST.
39. Remove the chamber slide cassette.

***Note:*** If using NEST chamber slides, the clamping mechanism and chamber can be decontaminated and sterilized using UV and 70% EtOH, and then reused with either glass slides or tissue-culture treated polystyrene slides for significant cost savings. Ensure that during UV sterilization, the surfaces are facing the light-source and not blocked by other surfaces. Ibidi and Thermo Scientific Lab-Tek chamber slides have been validated, but they are built using adhesive and cannot be reused.

40. Mount the slides using Fluoromont G and they are ready for bright-field or confocal imaging. Store them at 4°C or − 20°C for more extended storage.

## Expected outcomes

In this protocol, we propose a method for 3D organoid culturing of murine OE cells for up to 14 days. It can be performed with adult mice and requires neither transgenic mice nor purification of the starting material beyond microdissection of the appropriate olfactory region. Respiratory tissue contaminant will grow in this media, but not form olfactory organoids. The model supports neurogenesis and the generation of immature olfactory neurons. The described organoid model enables the in situ development of axonal projections in neurons, as organoids are attached to the growth substrate.

**Figure 12:**
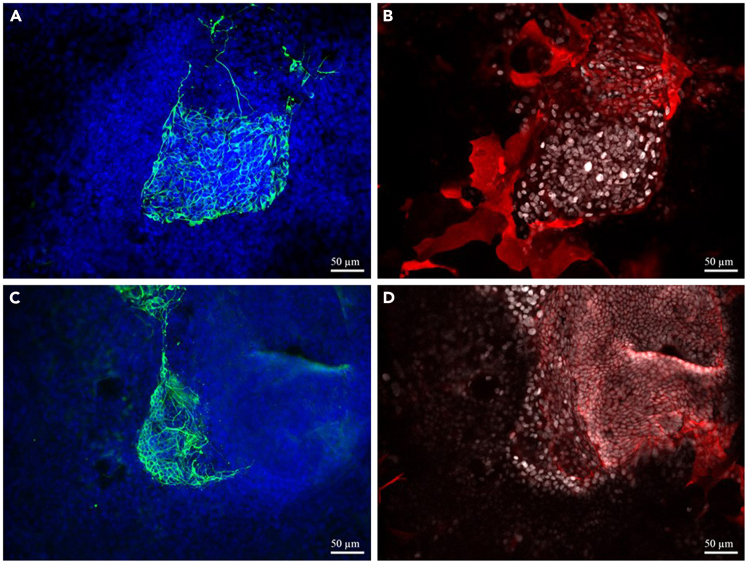
Immunofluorescence staining results Representative 20x bright-field images of organoids stained for TUJ1 (neuronal) and DAPI (nuclei) (A and C), and KRT14 (basal cell) and Sox2 (stem/progenitor cell) (B and D). Panels (A and B) use B27 supplement containing retinoic acid, while Panels (C and D) use B27 supplement without retinoic acid.

In a successful experiment, we obtained an average of 31 organoids per well by day 7 of organoid culture, with each organoid comprising an average of 49 Tuj1+ olfactory sensory neurons. Immunostaining should reveal organoids containing KRT5+/SOX2+ horizontal basal cell-like cells in close proximity to KRT5−/SOX2+ GBC-like cells, neuronally committed/neuron-producing downstream NEUROD1+ globose basal cell-like cells (KRT5−/SOX2+/NEUROD1+), and immature olfactory sensory neurons (iOSN) (KRT5−/SOX2−/Tuj+/GAP43+/UCHL1+). Furthermore, there should be Tuj1+/UCHL1+/GAP43+ neurites emanating from organoids (Figures 12A–12D, staining of culture with & w/o RA in B27 at day 7 and 14).

## Limitations

As the focus of this model for 3D organoid culture of murine OE is neurogenesis, the culture conditions are designed to be pro-neurogenic. Therefore, other cell types, such as sustentacular or duct/gland cells, are not generated.^1^ Although the model provides a neurogenic environment, mature olfactory sensory neurons are absent, which may be due to the lack of an olfactory bulb or other unknown signals or trophic factors.^1^ While the cultures survive dissociation and passaging for expansion, subsequent passages show significantly diminished organization capacity. Finally, the protocol is limited to mouse cells and cannot be replicated with human cells under these exact conditions.

## Troubleshooting

### Problem 1

Low cell count after dissociation.

### Potential solution

Ensure that the dissected sample is adequately minced prior to mixing it into the papain enzyme solution. Ideally, mince until a crunching sound from cutting bone/cartilage can no longer be heard. In addition, consider increasing papain incubation time by 5-10 min.

### Problem 2

Excessive gel artifacts in the culture.

### Potential solution

Cultrex has gelled prematurely, likely due to the initial seeding media warming up. Consider bringing an ice bucket into the tissue culture hood to keep the media cold.

### Problem 3

Organoids exist in culture but disappear after fixation.

### Potential solution

pH of the 4% PFA is critical for maintaining organoids on the slide. Re-adjust pH to within 7.2–7.4.

## Resource availability

### Lead contact

Further information and requests for resources and reagents should be directed to and will be fulfilled by the lead contact, Dr. Brian Lin (brian.lin@tufts.edu).

### Technical contact

For technical specifics on executing the protocol, Dr. Juliana Gutschow Gameiro (juliana.g.gameiro@gmail.com) will provide support to ensure its correct implementation.

### Materials availability

This study did not generate new unique reagents and materials.

### Data and code availability

• All data are available from the lead author upon request.
• This article does not report original code.

## Acknowledgments

J.G.G. was funded by the Coordenação de Aperfeiçoamento de Pessoal de Nível Superior – Brasil (CAPES) (finance code 001). J.V. was funded by the Sigrid Jusélius Foundation and Orion Research Foundation sr. This study was supported by the National Institutes of Health (Project NIDCD R21DC018681-01 and R01DC017869-03).

## Author contributions

Conceptualization, J.G.G. and B.L.; methodology, J.G.G., J.V., L.L., and B.L.; resources, J.G.G., J.V., and J.E.S.; writing – original draft, J.G.G., J.V., and L.L.; writing – review and editing, J.G.G., J.V., L.L., J.E.S., M.A.F., and B.L.; supervision, J.E.S., M.A.F., and B.L.

## Declaration of interests

J.E.S. is a co-founder of Rhino Therapeutics. B.L. is a consultant for Rhino Therapeutics. B.L. is a co-founder of Cellsor, Inc.
